# Comparison of the effects of triglyceride variability and exposure estimate on clinical prognosis in diabetic patients

**DOI:** 10.1186/s12933-022-01681-8

**Published:** 2022-11-15

**Authors:** Sung Min Koh, Se Hwa Chung, Yun Jin Yum, Se Jun Park, Hyung Joon Joo, Yong-Hyun Kim, Eung Ju Kim

**Affiliations:** 1 Department of Internal Medicine, Korea University Anam Hospital, Seoul, Republic of Korea; 2 Department of Biostatistics, Korea University College of Medicine, Seoul, Republic of Korea; 3 Division of Cardiology, Department of Internal Medicine, Korea University Anam Hospital, Seoul, Republic of Korea; 4 Department of Medical Informatics, Korea University College of Medicine, Seoul, Republic of Korea; 5 College of Medicine, Korea University Research Institute for Medical Bigdata Science, Korea University, Seoul, Republic of Korea; 6 Division of Cardiology, Department of Internal Medicine, Korea University Ansan Hospital, Ansan, Republic of Korea; 7 Division of Cardiology, Department of Internal Medicine, Korea University Guro Hospital, Seoul, Republic of Korea

**Keywords:** Triglyceride, Variability, Cumulative exposure, Major adverse event

## Abstract

**Background:**

Hypertriglyceridemia is an important feature of dyslipidemia in type 1 and type 2 diabetic patients and associated with the development of atherosclerotic cardiovascular disease. Recently, variability of lipid profile has been suggested as a residual risk factor for cardiovascular disease. This study compared the clinical impact of serum triglyceride variability, and their cumulative exposure estimates on cardiovascular prognosis in diabetic patients.

**Methods:**

A total of 25,933 diabetic patients who had serum triglyceride levels measured at least 3 times and did not have underlying malignancy, myocardial infarction (MI), and stroke during the initial 3 years (modeling phase) were selected from three tertiary hospitals. They were divided into a high/low group depending on their coefficient of variation (CV) and cumulative exposure estimate (CEE). Incidence of major adverse event (MAE), a composite of all-cause death, MI, and stroke during the following 5 years were compared between groups by multivariable analysis after propensity score matching.

**Results:**

Although there was a slight difference, both the high CV group and the high CEE group had a higher cardiovascular risk profile including male-dominance, smoking, alcohol, dyslipidemia, and chronic kidney disease compared to the low groups. After the propensity score matching, the high CV group showed higher MAE incidence compared to the low CV group (9.1% vs 7.7%, *p* = 0.01). In contrast, there was no significant difference of MAE incidence between the high CEE group and the low CEE group (8.6% vs 9.1%, *p* = 0.44). After the multivariable analysis with further adjustment for potential residual confounding factors, the high CV was suggested as an independent risk predictor for MAE (HR 1.19 [95% CI 1.03–1.37]).

**Conclusion:**

Visit-to-visit variability of triglyceride rather than their cumulative exposure is more strongly related to the incidence of MAE in diabetic patients.

**Supplementary Information:**

The online version contains supplementary material available at 10.1186/s12933-022-01681-8.

## Background

Significant portion of atherosclerosis progression is attributed to dyslipidemia [1–5]. In particular, lipid accumulation over a long duration of time is closely related to poorer cardiovascular outcomes and all-cause mortality [6–8]. Along with increased serum lipid levels, presence of type 1 or type 2 diabetes mellitus is also one of the major risk factors of atherosclerotic cardiovascular disease, which is a leading cause of death in diabetic patients [9, 10]. Therefore, presence of dyslipidemia together with diabetes mellitus may result in increased cardiovascular risk and bring poorer clinical outcomes [11, 12].

Importantly, dyslipidemia in type 2 diabetic patients is characterized by high serum triglyceride (TG) level, which is thought to lead to increased small dense low-density lipoprotein (LDL) particles. These small dense LDL particles are thought to be more atherogenic than other large-buoyant LDL particles [13, 14]. This suggests serum TG level may play an important role especially in diabetic patients. Previously, hypertriglyceridemia has been suggested to be an important cardiovascular risk factor [15–18]. And, it was reported that the higher serum TG level in diabetic patients, the higher the mortality rate [19]. However, there are also conflicting studies on the effect of hypertriglyceridemia on cardiovascular prognosis. Di Angelantonio et al. showed that mean level of serum triglyceride (TG) is not significantly associated with coronary heart disease [1]. Xia et al. also showed that increased serum TG level was associated with decreased all-cause mortality and cardiovascular mortality (so-called “TG paradox”) [20]. These conflicting findings suggest that the clinical significance of serum TG level may differ depending on various contextual circumstances.

Variability may be one major threat to disrupting the homeostasis of human health. For example, it has been well-known that higher variability on blood pressure or heart rate is associated with adverse clinical outcome [21]. For diabetic patients, glycemic variability has been reported to be associated with the increased risk of hypoglycemia, microvascular and macrovascular complications as well as mortality [22]. Blood glucose as well as TG are the representative biochemicals with high variability in diabetic patients [23]. Serum TG level is not only high in diabetic patients but is also highly variable because it is more affected by diet than other lipid components [24]. It has also been reported that postprandial TG variability is associated with renal impairment and microalbuminuria in diabetic patients [25]. These characteristics of TG in diabetic patients suggest that it is necessary to assess the TG measurements from the other aspects beyond simply high and low values in clinical application.

Previously, visit-to-visit variability in blood lipid (total cholesterol (TC), as well as LDL-cholesterol (LDL-C), high-density lipoprotein-cholesterol (HDL-C)) was suggested to correlate with clinical outcomes of a variety of groups, from the general population to those at high cardiovascular risk [26–32]. Regarding the diabetic patients, Wan et al. suggested that the increased variabilities of TG, LDL-C, and TC/HDL-C could be associated with the increased cardiovascular disease and mortality [33, 34]. Bardini G et al*.* also reported that serum TG variability is a predictor of incident microalbuminuria. These suggested that serum TG variability has an adverse effect on patients with diabetes mellitus. Nevertheless, whether cumulative accumulation of serum TG or its variability would be a more determining factor is still not clear. In this study, we investigated the clinical impact of serum TG variability and cumulative exposure estimate on major adverse events (MAE) specifically in diabetic patients.

## Methods

### Study design

This research was a multicenter retrospective cohort study using the Observational Medical Outcomes Partnership (OMOP) Common Data Model (CDM) database of three tertiary hospitals (Korea University Anam Hospital, Korea University Guro Hospital, and Korea Ansan Hospital) in Korea. The Observational Health Data Sciences and Informatics collaboration provides the OMOP CDM schema, which is used to standardize the electronic health records of hospitals into the OMOP CDM database [35]. In Korea, the ICD-10 code system is used for disease classification, and OMOP-CDM provides unique concept IDs mapped to this code. Thus, the data were analyzed using the OMOP-CDM concept ID, which was mapped to the ICD-10 code. The detailed OMOP-CDM concept IDs are provided in the supplementary data (Additional file 1: Table S1). The OMOP-CDM data of the present study was extracted through direct querying.

**Fig. 1:**
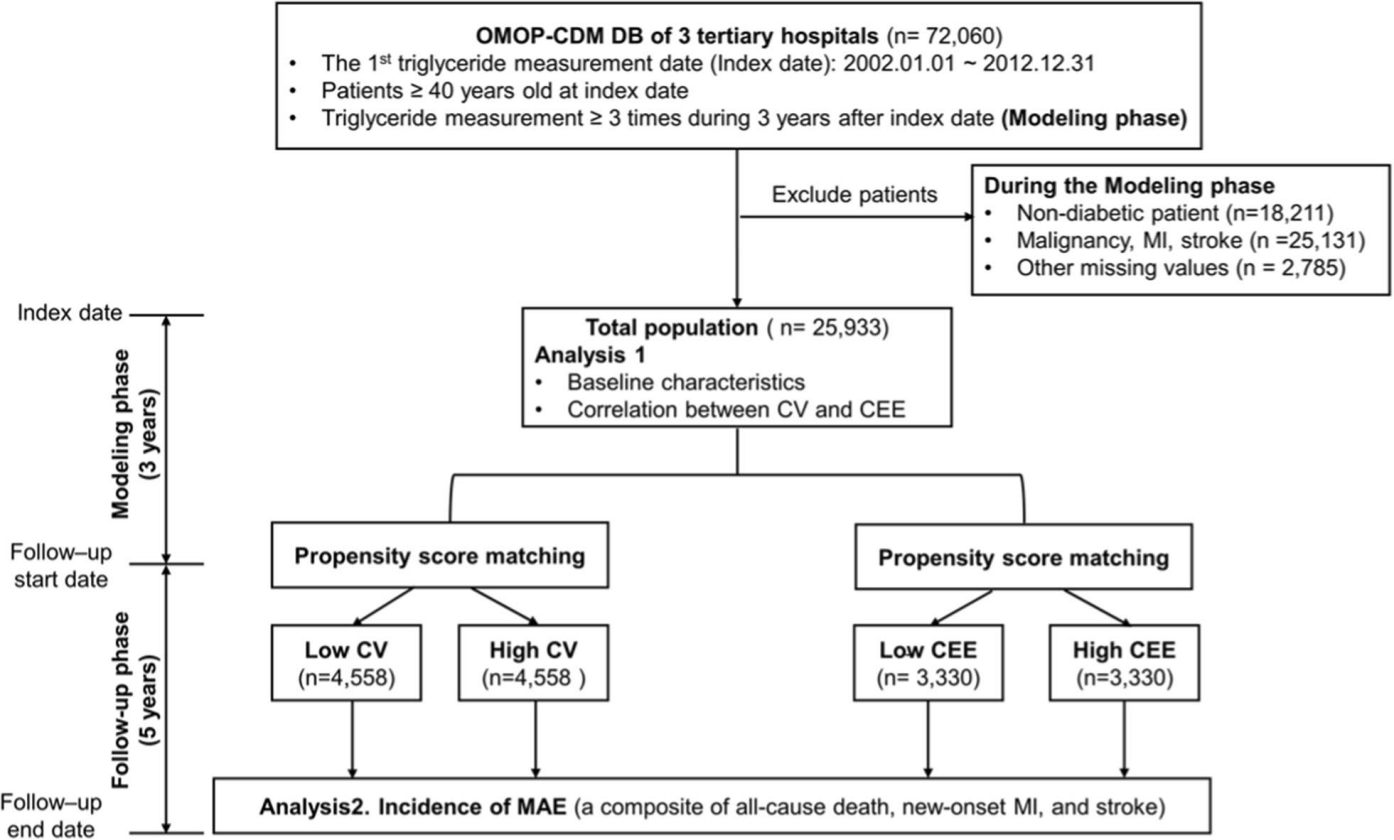
Study scheme. *CDM* common data model, *CEE* cumulative exposure estimate, *CV* coefficient of variation, *DB* database, *MAE* major adverse event, *MI* myocardial infarction, *OMOP* Observational Medical Outcomes Partnership

For selection of the study population, 72,060 patients aged 40 years and above, whose 1st serum TG level measurement was taken between January 2002 and December 2012 and measured 3 or more times during the initial 3 years were selected (Fig. 1). From these 3-eyar TG values, variability and cumulative exposure were estimated (Modeling phase). Patients who were not diabetic were excluded from the study (n = 18,211). Patients with already known malignancy, myocardial infarction (MI), and stroke at baseline were excluded as well (n = 25,131). Patients with missing values including serum creatinine whose presence of chronic kidney disease (CKD) cannot be determined were also excluded (n = 2785). Finally, 25,933 patients were analyzed for this study.

The study was approved by the Review Board of the three institutes. Written informed consent was waived due to the retrospective study design and use of anonymized data with minimal risk to the study participants. The study complied with the principles of the Declaration of Helsinki. This study adopted the results of routine standard laboratory tests. Blood sampling for routine standard laboratory tests was performed during daytime after overnight fasting. Serum levels of lipid profile were measured using the homogeneous enzymatic colorimetric assay.

### Triglyceride exposure estimate and variability

To define the cumulative exposure estimate (CEE) and coefficient of variation (CV) of TG, we inspected the 3 year TG measurements of the patients. For CEE, we used a cubic spline-based mixed effects model (linear mixed-effects model with cubic spline) to account for the unbalanced distribution of measurements over an individual and for the flexibility in modelling non-linear variation over time. Random intercepts and random slopes were also included in the model to consider individual variations in intercepts and individual variability in serum TG levels, respectively. The area under the curve for the model of each patient was calculated as the CEE. The coefficient of variation (CV) was used as the variability index which was calculated as $100\times \frac{\sigma }{\mu }$, where $\sigma$ is the standard deviation and $\mu$ is the mean of the serum TG levels.

### Definitions and study endpoint

Hypertension was defined as systolic blood pressure ≥ 140 mmHg, or diastolic blood pressure ≥ 90 mmHg, being on anti-hypertensive medication, or having OMOP-CDM concept ID for hypertension. Diabetes mellitus was defined as HbA1c ≥ 6.5%, or being on antidiabetic medication, or having OMOP-CDM concept ID for diabetes mellitus or fasting plasma glucose ≥ 126 mg/dL. The presence of chronic kidney disease was defined based on the Modification of Diet in Renal Disease [36] equation with glomerular filtration rate being less than 60 mL/min/1.73 m^2^. Dyslipidemia was defined as having OMOP-CDM concept ID for dyslipidemia or serum TC ≥ 240 mg/dL, LDL-C ≥ 160 mg/dL, TG ≥ 200 mg/dL, or HDL-C < 40 mg/dL or taking lipid lowering drug. Patients taking medications including anti-hypertensive drugs, statins, TG-lowering drugs including fenofibrates and omega-3-fatty acids, antiplatelets and anticoagulants were defined as those who were prescribed with the medications with proportion of days covered ≥ 50% during the modeling phase.

The primary endpoints of this study were 5 year MAE, a composite of all-cause death, new-onset MI or stroke. MI was defined as having OMOP-CDM concept ID for MI or serum creatinine kinase MB (CK-MB) level greater than upper limit of normal with a rising and/or falling pattern. Stroke was defined as having the corresponding OMOP-CDM concept ID or having acute, sub-acute or recent cerebral infarction findings on brain MRI. Survival time was considered as the time from the follow-up start date to the date of MAE, or until the end of follow-up, whichever was first.

### Statistical analysis

Categorical data were represented as number (%) and continuous data are shown as the mean ± SD in baseline characteristics. Chi-square test and Student’s t-test were used to compare the categorical variables and continuous variables between groups. Pearson’s correlation coefficient was used to assess whether the effects of TG CEE and CV were independent of each other. A logistic regression model was used to calculate the propensity score for each patient of the high CV group and the low CV group and matching was performed using caliper 0.25 (1:1). Covariates included age, sex, alcohol, smoke, hypertension, dyslipidemia, CKD, creatinine, insulin use, TG-lowering drug, antiplatelet drug, anticoagulant, HbA1c, high-sensitivity C-reactive protein (hsCRP), TC, LDL-C, HDL-C, TG, number of TG measurement, and interval between TG measurement. Standardized mean differences of < 0.15 was considered to indicate good balance. The same method was applied to match the high CEE group and the low CEE group. Baseline serum TG level was highly correlated with CEE. Since the CEE groups can be easily estimated from the baseline TG level, including the baseline TG level in the propensity score matching variables could obscure some of the target effects. Thus, baseline TG level was excluded from the covariates when matching the propensity scores of the high CEE group and the low CEE group. The MAE event probabilities were calculated using Kaplan–Meier curves and compared by estimating the log-rank test. A multivariable Cox proportional hazards regression analysis was performed to confirm the prognosis of MAE between the two groups after additional adjustment of variables that have not been adjusted even after matching. The variables with a p-value less than 0.1 of the Chi-square test or Student’s t-test in baseline analysis after the propensity score matching were selected for the further adjustment. Clinically important variables that could affect serum TG level were also selected. Finally, dyslipidemia, TG lowering drugs, anticoagulants, creatinine, hsCRP, TG, number of TG measurement were additionally selected for the further adjustment for the CV groups. Dyslipidemia, TG lowering drugs, TC, HDL-C were additionally selected for the further adjustment for the CEE groups. The proportional hazards assumption for the variables in the models was assessed by inspecting Schoenfeld residuals. All analyses were performed using SAS (version 9.4; SAS Institute Inc., Cary, NC, USA) and R program (version 4.1.2). Cubic spline modeling was performed using the R program with the ‘*lme4*’ package, and plots were drawn using the R program with the ‘*ggplot2*’ and ‘*survminer*’ packages. Propensity score matching was performed using the ‘*proc psmatch*’ procedure in SAS program.

## Results

### Baseline characteristics

The high CV group and the low CV group were divided based on the median coefficient of variation for serum TG level of the total population (n = 25,933). Comparison of the baseline characteristics between the high CV group and the low CV group are shown in Table 1. The high CV group were younger and had lower rates of taking anti-hypertensive medications and lower mean LDL-C level. However, except for these, all other variables showed higher cardiovascular risk profile in the high CV group compared to the low CV group. The high CEE group and the low CEE group were divided based on the median cumulative exposure estimate for serum TG level of total population. Comparison of the baseline characteristics between the high CEE group and the low CEE group are shown in Table 2. The high CEE group were younger and had lower rates of insulin use. Except for these, all other variables showed higher cardiovascular risk profile in the high CEE group compared to the low CEE group.

**Table 1:** Baseline characteristics between high CV and low CV groups

Variables	Low CV (n = 12,967)	High CV (n = 12,966)	*p*
Age (years)	63.6 ± 10.4	61.5 ± 10.2	< .01
Male (n, %)	6353 (49.0)	7108 (54.8)	< .01
BMI (kg/m^2^)	25.1 ± 10.7	25.0 ± 7.3	0.45
Current smoker (n, %)	757 (5.8)	1078 (8.3)	< .01
Alcohol drinking (n, %)	854 (6.6)	1265 (9.8)	< .01
Systolic BP (mmHg)	120.3 ± 10.1	120.6 ± 10.1	0.10
Diastolic BP (mmHg)	74.2 ± 5.6	74.6 ± 5.6	< .01
Hypertension (n, %)	8196 (63.2)	8104 (62.5)	0.24
Anti-hypertensive drug (n, %)	7108 (54.8)	6899 (53.2)	0.01
Dyslipidemia (n, %)	8697 (67.1)	9767 (75.3)	< .01
Statin (n, %)	5634 (43.5)	5555 (42.8)	0.33
TG-lowering drug (n, %)	163 (1.1)	640 (4.9)	< .01
Chronic kidney disease (n, %)	2760 (21.3)	2928 (22.6)	0.01
Insulin use (n, %)	3099 (23.9)	3685 (28.4)	< .01
Antiplatelet drug (n, %)	4753 (36.7)	4678 (36.1)	0.34
Anticoagulant (n, %)	227 (1.8)	245 (1.9)	0.40
Glucose (mg/dL)	134.6 ± 32.8	138.9 ± 36.2	< .01
HbA1c (%)	7.1 ± 1.2	7.2 ± 1.2	< .01
Creatinine (mg/dL)	1.15 ± 1.03	1.32 ± 1.46	< .01
hsCRP (mg/dL)	3.1 ± 2.9	3.4 ± 3.0	< .01
Total cholesterol (mg/dL)	171.7 ± 31.1	174.0 ± 32.9	< .01
LDL-cholesterol (mg/dL)	101.3 ± 26.5	99.9 ± 28.3	< .01
HDL-cholesterol (mg/dL)	48.2 ± 11.8	46.5 ± 11.4	< .01
Triglyceride (mg/dL)	140.7 ± 70.5	180.7 ± 99.3	< .01
TG-CV (%)	20.8 ± 6.5	46.6 ± 15.9	< .01
TG-CEE (arbitrary unit)	5061.8 ± 1918.3	6173.0 ± 2679.2	< .01
Number of TG measurement	5.9 ± 3.6	6.6 ± 4.2	< .01
Interval between TG measurement (day)	221.5 ± 131.7	203.5 ± 126.2	< .01

Categorical variables in n (%) and continuous variables in mean ± standard deviation

*BMI* body mass index, *BP* blood pressure, *hsCRP* high-sensitivity C-reactive protein, *LDL* low density lipoprotein, *HDL* high density lipoprotein, *TG-CV* coefficient of variation for serum TG level, *TG-CEE* cumulative exposure estimates for serum TG level

**Table 2:** Baseline characteristics between high CEE and low CEE groups

Variables	Low CEE (n = 12,967)	High CEE (n = 12,966)	*p*
Age (years)	63.7 ± 10.4	61.4 ± 10.2	< .01
Male (n, %)	6392 (49.3)	7069 (54.5)	< .01
BMI (kg/m^2^)	24.3 ± 6.2	25.9 ± 11.1	< .01
Current smoker (n, %)	837 (6.5)	998 (7.7)	< .01
Alcohol drinking (n, %)	973 (7.5)	1146 (8.8)	< .01
Systolic BP (mmHg)	120.1 ± 10.3	120.9 ± 9.9	< .01
Diastolic BP (mmHg)	74.0 ± 5.7	74.9 ± 5.4	< .01
Hypertension (n, %)	7850 (60.5)	8450 (65.2)	< .01
Anti-hypertensive drug (n, %)	6729 (52.2)	7238 (55.8)	< .01
Dyslipidemia (n, %)	7628 (58.8)	10,836 (83.6)	< .01
Statin (n, %)	5466 (42.2)	5723 (44.1)	0.01
TG-lowering drug (n, %)	73 (0.6)	703 (5.4)	< .01
Chronic kidney disease (n, %)	2645 (20.4)	3043 (23.5)	< .01
Insulin use (n, %)	3534 (27.3)	3250 (25.1)	< .01
Antiplatelet drug (n, %)	4758 (36.7)	4673 (36.0)	0.28
Anticoagulant (n, %)	249 (1.9)	223 (1.7)	0.23
Glucose (mg/dL)	134.4 ± 33.9	139.1 ± 35.2	< .01
HbA1c (%)	7.0 ± 1.2	7.2 ± 1.2	< .01
Creatinine (mg/dL)	1.22 ± 1.30	1.25 ± 1.23	0.12
hsCRP (mg/dL)	3.3 ± 3.0	3.3 ± 2.9	0.87
Total cholesterol (mg/dL)	164.1 ± 30.0	181.7 ± 31.6	< .01
LDL-cholesterol (mg/dL)	96.2 ± 25.8	105.1 ± 28.4	< .01
HDL-cholesterol (mg/dL)	50.2 ± 12.5	44.5 ± 10.0	< .01
Triglyceride (mg/dL)	102.0 ± 23.8	219.4 ± 90.5	< .01
TG-CV (%)	29.5 ± 14.4	37.9 ± 19.7	< .01
TG-CEE (arbitrary unit)	3984.9 ± 667.8	7250.0 ± 2387.3	< .01
Number of TG measurement	6.0 ± 3.9	6.5 ± 4.0	< .01
Interval between TG measurement (day)	222.8 ± 131.8	202.2 ± 126.0	< .01

Categorical variables in n (%) and continuous variables in mean ± standard deviation

*BMI* body mass index, *BP* blood pressure, *hsCRP* high-sensitivity C-reactive protein, *LDL* low density lipoprotein, *HDL* high density lipoprotein, *TG-CV* coefficient of variation for serum TG level, *TG-CEE* cumulative exposure estimates for serum TG level

### Correlation between triglyceride CV and CEE

**Fig. 2:**
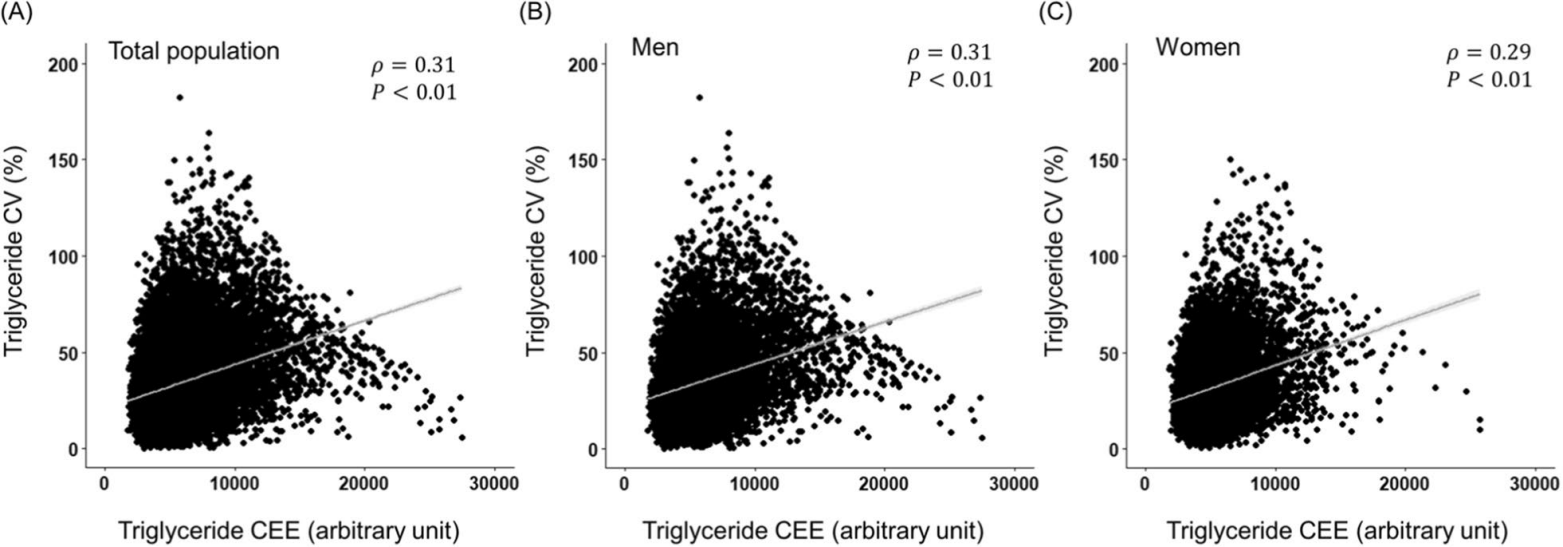
Correlation between CV and CEE in diabetic patients. **A**Total population, **B** men and **c** women

Correlation between CV and CEE for the serum TG level was investigated to explore how independent they were as clinical factors. There was a weak positive correlation between the CV and the CEE (ρ = 0.31, *p* < 0.01; Fig. 2). The correlation was consistent regardless of sex.

### Cumulative incidence of major adverse events

To evaluate the effect of serum TG level more accurately in diabetic patients, the high CV group and the low CV group were reselected through propensity score matching (n = 4558 in each group). The high CEE group and the low CEE group were also reselected by propensity score matching (n = 3330 in each group). The baseline characteristics of the study populations after propensity score matching are shown in Additional file 1: Table S2 and Table S3, respectively.

**Fig. 3:**
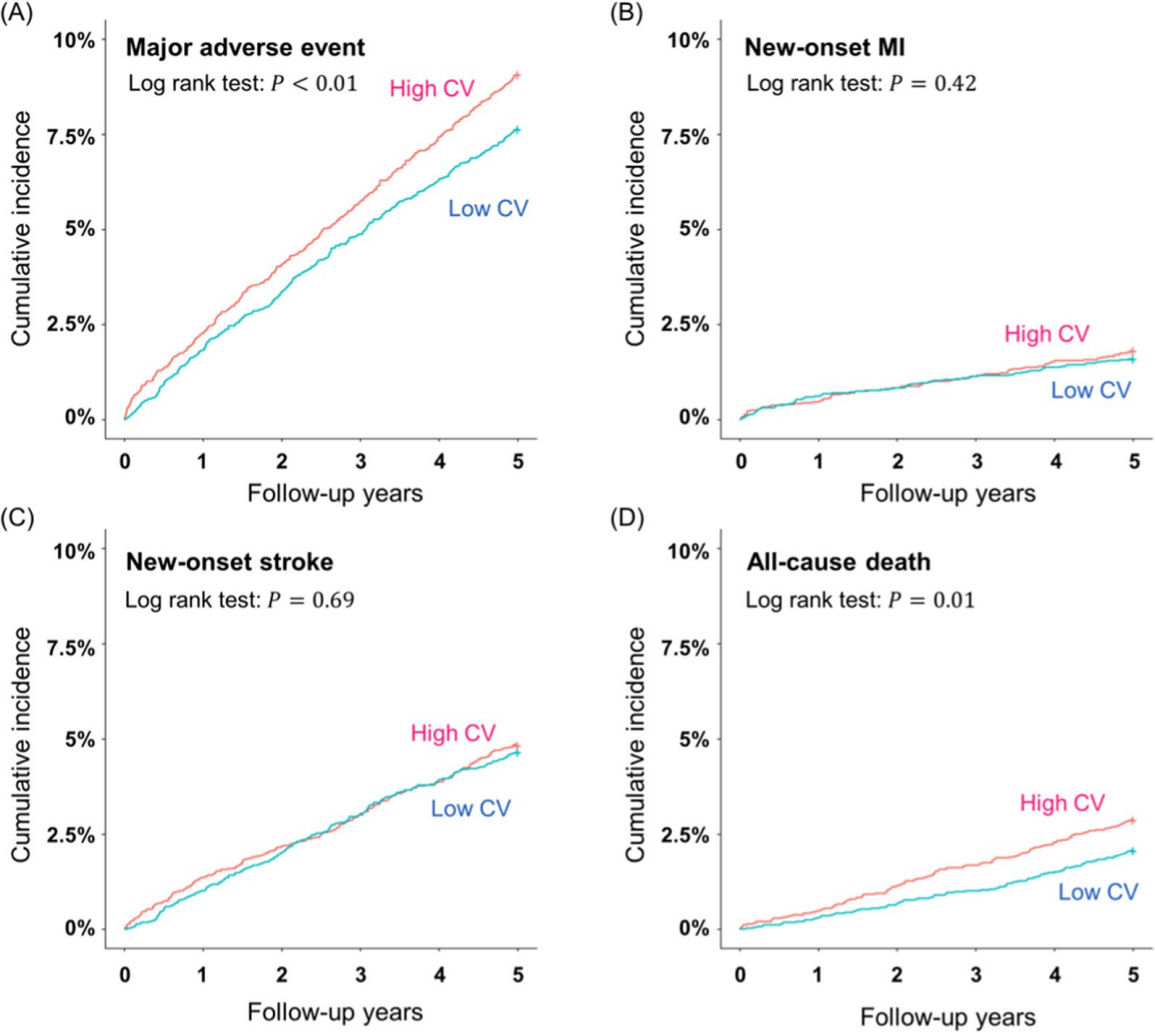
Kaplan–Meier plot of cumulative incidences of MAE, new-onset MI, stroke and all-cause death between the high CV group and the low CV group **A** MAE, (**B**) new-onset MI, (**C**) new-onset stroke, and (**D**) all-cause death. *MAE* major adverse events, *MI* myocardial infarction, *CV* coefficient of variation

**Fig. 4:**
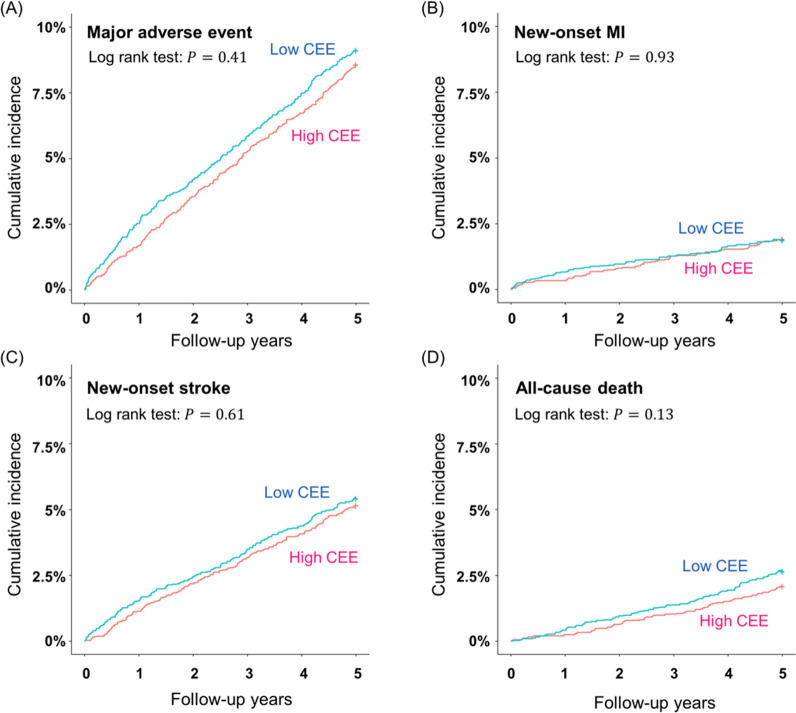
Kaplan–Meier plot of cumulative incidences of MAE, new-onset MI, stroke and all-cause death between the high CEE group and the low CEE group (**A**) MAE, (**B**) new-onset MI, (**C**) new-onset stroke, and (**D**) all-cause death. *MAE* major adverse events, *MI* myocardial infarction, *CEE* cumulative exposure estimate

The number of cases and incidence rates of major adverse events, including all-cause death, new-onset MI, and stroke in the study populations after propensity score matching are presented in Table 3. The median follow up was 1095 days. The incidence rates of MAE and all-cause deaths were significantly greater in the high CV group compared to that of the low CV group (9.1% vs. 7.7% and 2.9% vs. 2.1%, respectively; *p* = 0.01). No statistically significant differences were observed between the high CEE group and the low CEE group. The Kaplan–Meier curves showed the consistent results (Figs. 3 and 4).

**Table 3:** Comparison of the incidence of MAE, new-onset MI, stroke, and all-cause death among the groups after propensity score matching

	Low CV (n = 4558)	High CV (n = 4558)	*p*	Low CEE (n = 3330)	High CEE (n = 3330)	*p*
MAE	349 (7.7)	414 (9.1)	0.01	304 (9.1)	286 (8.6)	0.44
New-onset MI	73 (1.6)	83 (1.8)	0.42	63 (1.9)	64 (1.9)	0.93
New-onset stroke	213 (4.7)	221 (4.9)	0.69	181 (5.4)	172 (5.2)	0.62
All-cause death	95 (2.1)	132 (2.9)	0.01	89 (2.7)	70 (2.1)	0.13

*p* is the value of the log-rank test

Values are presented as number of incidence (%)

*MAE* major adverse event, *MI* myocardial infarction

### Multivariable Cox regression analysis

Multivariable Cox regression analysis was performed to validate the significance of CV and CEE as an independent risk predictor for MAE, new-onset MI, stroke, and all-cause death (Table 4). As a result, the high CV for TG was suggested as an independent predictor for MAE and all-cause death (HR 1.19 [95% CI 1.03–1.37] for MAE, HR 1.37 [95% CI 1.05–1.78] for all-cause death).

**Table 4:** Multivariable Cox regression models for MAE, new-onset MI, stroke, and all-cause death

	High CV vs Low CV (reference)	High CEE vs Low CEE (reference)
MAE	1.19 (1.03–1.37)	0.93 (0.79–1.09)
New-onset MI	1.11 (0.81–1.53)	0.99 (0.70–1.39)
New-onset stroke	1.03 (0.86–1.25)	0.94 (0.76–1.16)
All-cause death	1.37 (1.05–1.78)	0.80 (0.59–1.09)

Values are presented as hazard ratio (95% confidence interval). Adjusted variables were dyslipidemia, TG lowering drug, anticoagulant, creatinine, hsCRP, TG, number of TG measurement in CV group. In CEE group, dyslipidemia, TG lowering drug, TC, HDL-C were adjusted

*MI* myocardial infarction, *MAE* major adverse event.

## Discussion

The main findings of the study were as follows: 1) Similar to the diabetic patients with high cumulative TG exposure (high CEE group), the patients with the high TG variability (high CV group) had a higher cardiovascular risk profile including male-dominance, smoking, alcohol, dyslipidemia, and chronic kidney disease compared to those with the low TG variability (low CV group). 2) TG variability and its CEE showed only weak correlation, suggesting that the high CV group would have their own characteristics and clinical impacts different from the high CEE group. 3) Finally, unlike the high CEE, which was not associated with MAE, the high CV was suggested as an important predictor of MAE, especially mortality.

Here we showed TG variability rather than their cumulative exposure is more important for prediction of major adverse clinical events. Exactly how variability of serum TG level affect clinical events remains largely unknown. Nordestgaard and Varbo suggested TG may serve as an important surrogate marker for raised remnants rich in atherosclerotic plaque-forming cholesterol [18, 37–39]. Clark et al. suggested that greater variability in atherogenic lipoprotein levels is significantly associated with coronary atheroma volume progression and clinical outcomes [26]. While this study did not analyze the effect of triglyceride on its own, it may be equivalently explained that failure to maintain one’s homeostatic ability to cope with atherosclerotic plaque-forming cholesterol remnants may be reflected by increased TG variability, leading to further progression atheroma formation in vessels.

Prior studies have suggested that long-term accumulation of serum lipids contribute to increased risk of cardiovascular diseases [6–8]. Among studies that evaluated the effect of TG, some studies showed mean TG level is not associated with cardiovascular disease events, or even inversely correlated [1, 20]. These conflicting results may be possibly put together by TG variability. Waters et al. and Wan et al. suggested that TG variability was significantly associated with greater cardiovascular risk factors and prognosis in patients with coronary artery disease or diabetes mellitus [29, 34]. These studies suggested that TG variability is associated with atherosclerotic cardiovascular disease, but they mainly focused on serum LDL, HDL, TC, and non-HDL levels, but not on TG [26–28, 30].

On the contrary, Wang et al. failed to show that TG variability is associated with all-cause and cardiovascular mortality in diabetic patients [40]. This difference may be attributed to the difference of study populations among the studies. The study of Wang et al. showed higher mortality rate of 17.4% for 6.4-year follow-up period, but the mortality rate in our study was less than 3% for 5-year follow-up period. In addition, the study of Wang et al. analyzed the impact of 10% increase in TG coefficient of variation. On the other hand, the difference of TG coefficient of variation between the high CV group and the low CV group was 23.4% in our study (Additional file 1: Table S2). It suggested that TG variability in the study of Wang et al. might be underestimated compared to our study.

Importantly, it is difficult to study a TG-independent role for clinical prognosis because TG is associated with the other lipid profiles and the occurrence of cardiovascular risk factors including diabetes and CKD. High TG levels rather than high LDL-C or low HDL-C have been suggested as an independent risk factor for incident diabetes [41]. High TG variability was also predictive of incident diabetes [29]. In addition, the incidence of kidney disease was associated with high TG variability in diabetic patients [42]. While TG increase itself may affect cardiovascular risk factors, our study tried to analyze the independent effect of TG variability by conducting propensity score matching and further adjusting the other major confounding variables among patient groups. Results showed that variability in serum TG level was an independent risk factor for major adverse events.

Whether clinical intervention to reduce serum TG levels would also help to improve cardiovascular prognosis still remains unclear and more randomized intervention trials are necessary [18]. Several studies showed TG-lowering drugs including fibrates and niacin helped to decrease cardiovascular events and all-cause mortality [43–51]. Interestingly, Bangalore et al. suggested that the administration of higher doses of statin decreased the extent of lipid variability, especially LDL-C, and eventually lowered the risk of cardiovascular events [32]. Similarly, it would be important to see if TG-lowering drugs that are known to lessen cardiovascular risks take the cardiovascular protective effect by lowering TG variability in the future studies. Although there is still no relevant treatment option for TG variability, but more caution would be helpful for the diabetic patient with high TG variability in clinical practice considering the high cardiovascular risk.

The present study had several limitations. First, there may be selection biases. The study population was confined to patients from three tertiary medical centers. Patients may tend to have more aggressive and dedicated care for their accompanying illnesses. More than 40% of the study population took statins (the highest proportion compared to the other studies), suggesting that cardiovascular risk driven high lipid levels is likely to be significantly attenuated. Nevertheless, high CV was consistently presented as an independent predictor for the occurrence of major adverse events in the analysis of the full sample population before propensity score matching (Additional file 1: Table S4) and regression analysis with various covariates (Additional file 1: Table S5 and Table S6). It suggests the robustness of this study result under different situations. Second, the study included the patients on omega-3 supplements and fibrates. Their TG variability may be affected by TG lowering drugs. Although their proportion was only 3% in our study, the actual number of patients taking omega-3 supplements might be much higher considering that omega-3 supplements can be easily obtained at pharmacies without a prescription. Third, our study analyzed only TG rather than the other lipid components. Although analysis on the other lipid components deepen the comprehensive insight regarding lipid variability in diabetic patients, we would like to focus on TG considering the unique characteristics of TG in diabetes. Finally, causality between TG variability and prognosis was not clear due to the retrospective nature of the study. Despite these limitations, novelty of the present study is that it was the first long-term observational study to compare the effects of TG variability and its cumulative exposure on the incidence of major clinical adverse events including mortality in diabetic patients.

In conclusion, diabetic patients with high TG variability had high cardiovascular risk profile. The significance of TG variability as an independent risk predictor for major adverse clinical event outweighed that of its cumulative exposure estimates.

## Supplementary Information

**Additional file 1: Table S1.** OMOP-CDM concept ID. **Table S2.** Baseline characteristics between high CV and low CV groups after the propensity score matching. **Table S3.** Baseline characteristics between high CEE and low CEE groups after the propensity score matching. **Table S4.** Incidence of endpoint in the full population before propensity score matching. **Table S5. **Cox regression analysis for TG CV in the full population before propensity score matching. **Table S6. **Cox regression analysis for TG CEE in the full population before propensity score matching.

**Additional file 2.** This file contains files for the analysis code of R and SAS programs used in this study.

## Data Availability

Under Korean law, personal health information cannot be taken out of the country without the consent of the study subjects. This study is a retrospective study that exempts the consent of the study subjects, and it is impossible to export the study dataset abroad.

## Abbreviations

CDM: Common data model
CEE: Cumulative exposure estimate
CI: Confidence interval
CKD: Chronic kidney disease
CV: Coefficient of variation
HDL-C: High-density lipoprotein-cholesterol
HR: Hazard ratio
hsCRP: High-sensitivity C-reactive protein
LDL-C: Low density lipoprotein-cholesterol
MAE: Major adverse event
MI: Myocardial infarction
OMOP: Observational medical outcomes partnership
TC: Total cholesterol
TG: Triglyceride
