# Supplementary material for: Comparison of the effects of triglyceride variability and exposure estimate on clinical prognosis in diabetic patients
Source: Cardiovasc Diabetol. 2022 Nov 15;21:245. doi: 10.1186/s12933-022-01681-8 (PMC9667663; doi:10.1186/s12933-022-01681-8)
Supplement: Supplementary file 1 — Additional file 1: Table S1. OMOP-CDM concept ID. Table S2. Baseline characteristics between high CV and low CV groups after the propensity score matching. Table S3. Baseline characteristics between high CEE and low CEE groups after the propensity score matching. Table S4. Incidence of endpoint in the full population before propensity score matching. Table S5. Cox regression analysis for TG CV in the full population before propensity score matching. Table S6. Cox regression analysis for TG CEE in the full population before propensity score matching. [file 12933_2022_1681_MOESM1_ESM.docx]

**Supplementary Data**

**Supplementary table 1.** OMOP-CDM concept ID

| **Condition** | **Concept_id** |
| --- | --- |
| SBP | 4152194 |
| DBP | 4154790 |
| Hypertension | 320128,4028741,4289933,314378,319034,443919,201313,439696,439695,  439694,195556,317895,443771,4110948,4028806,45769159,319826,4108213 |
| Anti-hypertensive drug | 1332527, 21141332, 974702, 1332497, 1337103, 1351559, 1351583, 1351587, 1321637, 1332494,19122209, 1332499, 40235487, 40235491, 42960852, 42960868, 974473, 974474, 19112606, 40167202, 42969135,  42960725, 42960735, 19023454, 19023453, 19050220, 19022242, 19022241,  19073094, 42950866, 42950863, 42950869, 42969118, 42969112, 42969115,  42969109, 42969102, 42969106, 42950421, 42950354, 42950393, 1332525,  40171917, 40171863, 40171884, 1308851, 19096678, 19096677, 1308874,  42950300, 1592858, 40174776, 21030573, 21097731, 19058101, 43275607,  42960860, 21601761, 42960864, 42925749, 42925752, 42925744, 40171661,  1337070, 40163142, 21070867, 42961744, 42961740, 1332419, 19073093, 19078080, 19078101, 40184187, 40184217, 19106542, 19106543, 36883910,  40184184, 19081025, 19074672, 1340161, 19074673, 42959999, 42960002,  42969040, 42969049, 42969067, 42969022, 42969031, 42969058, 42707639,  42707641, 42959791, 42959787, 40165789, 40165762, 19076924, 1353818,  19011549, 42963311, 42960018, 42960013, 42955514, 42955431, 19127433,  19127434, 19080128, 40185304, 40185276, 42948648, 42948651, 42948654,  42932547, 42932541, 42932544, 42932538, 44507582, 19106593, 19106594,  42929938, 42929951, 42929931, 1309071, 42801011, 42801015, 42952826, 19019309, 1318860, 19112979, 19101807, 43291777, 42960005, 42960008, 1305450, 1328585, 1328689, 19133612, 19133613, 46287343, 1353780, 42960035, 42960027, 42960031, 40163753, 40163760, 40174811, 19101751, 19101750, 21058150, 1326020, 19022948, 19022949, 19017656, 19015804, 19011548, 19134566, 1318859, 19112981, 1319942, 1319943, 19020063, 19113063, 40167843, 40167849, 40167852, 19102491, 42969132, 42969141, 42969126, 42969138, 42969129, 42969123, 19127432, 19096740, 19102171, 40069686, 19096752, 40163271, 40163275, 21041304, 21056210, 42968999, 42968981, 42968990, 42969008, 19102170, 1334461, 1334492, 42972637, 42972640, 42972631, 42972634, 42969082, 42969085, 42969088, 42969091, 42969094, 42969097, 19121182, 1332495, 35604949, 35604953, 35604961, 40224166, 40224172, 40224175, 42930395, 42930392, 974642, 19028935, 40165261, 40165245, 19028936, 42959698, 19107180, 974447, 19101748, 1353820, 42969152, 42969149, 42969157, 42969154, 42938510, 42938516, 42938513, 40171905, |
| Dyslipidemia | 437530, 437827, 440360, 438720, 437521, 432867, 435516, 437530, 4047784 |
| Statin | 19019116, 40175390, 40175394, 40175400, 41048773, 1539411, 1526479, 1539469, 1539407, 44506638, 44506641, 19112569, 19077499, 1545997, 19123592, 1545996, 1332497, 42972637, 19122209, 1545959, 43527029, 42972640, 42972631, 42972634, 43527032, 1332494, 1332499, 2057677, 2057662, 2057670, 2057647, 2057655, 40165245, 40165261, 40165253, 42969173, 42969291, 42969232, 42969040, 42969067, 42969132, 42969022, 42969141, 42969058, 42968976, 42969049, 42969129, 42969031, 2063250, 42969118, 42969135, 42969126, 42969138, 42969102, 42969085, 42969082  , 42969112, 42969109, 2063242, 42969008, 42969149, 42969154, 2063246, 42968999, 42969106, 2064553, 42968981, 2064586, 42969152, 2063264, 2063260, 42969157, 42969115, 2065490, 2064498, 42969094, 2064532, 2066023, 2066020, 2066024, 2065486, 42969088, 42969091, 2066021, 2063256, 2065482, 42968990, 2063252, 40165642, 40165646, 40165638, 42932538, 42932541, 42932547, 42932544, 2054845 |
| Tg-lowering drug | 19101756, 1551838, 19077244, 44506638, 44506641, 2054845,19022958  19121242,42968976,35143166 |
| Insulin | 19058398, 1596972, 19135264, 19078603, 19078559, 46234239  19078558, 19078552, 35602725, 42902356, 46221558, 43518492  42902587, 42902742, 42902821, 42902945, 41348912, 41349148,  41348682, 43275300, 42921644, 42921712, 42921721, 43297029,  46233971, 46233974, 46234047, 46234050, 46234234, 46234237 |
| Antiplatelet | 19103854, 42483115, 42949815, 19021575, 1112896, 1113143, 1718409, 19075601, 42949815, 40241188, 46287538, 40163724, 1350311, 1350332, 42926308,42926360,42935771 |
| Anticoagulant | 40163524, 40163554, 40228154, 35606208, 43013030, 43013026,  40244444, 40244448, 40241333, 45892856, 45892860, 45892850 |
| Glucose | 3004501 |
| HbA1c | 3007263 |
| Creatinine | 3016723, 3051825 |
| CK-MB | 3005785 |
| hsCRP | 3010156 |
| Total cholesterol | 3027114 |
| LDL-cholesterol | 3028437 |
| HDL-cholesterol | 3007070 |
| Triglyceride | 3022192 |
| MI | 434376, 438438, 438170, 438447, 441579, 312327, 436706, 4051874, 4270024,4108677, 4108218, 4108217 |
| Stroke | 4108952, 4078446, 4077958, 4077959, 4077201, 4111708, 432923, 4120104, 4049659, 4176892, 4218781, 4319328, 4326561, 4110185, 4110186, 43530727, 42535426, 436430, 4110189, 4110190, 46270031, 4110192, 4108356, 762933, 4111714, 443454, 373503, 374055, 437306, 4108357, 4112020, 4338523,  381591, 4045737, 4045738, 4046360, 4108360, 4110194, 4110195, 4111710,  4111711, 4301259, 379778, 443605, 4046089, 4046090, 4047747, 35610098 |
| Diabetes mellitus | 443732, 4099216, 443731, 443729, 37016354, 43530689, 37017432, 45769905, 45769906, 4193704, 4226121, 45757499, 43530656, 201530, 43530690, 45757277, 43530685, 36717156, 37016768, 4222415, 37016349, 443734, 4228443, 45757363, 37018728, 4063043, 4198296, 4129519, 4196141, 43531010, 4140466, 443412, 4224709, 4225055, 435216, 37017431, 36715571, 4295011, 45769904, 201254, 318712, 200687, 4222553, 377821, 42538169, 4224254, 45763583, 201531, 37016767, 45763584, 45757507, 37018566, 4227210, 37017429, 40484648, 45769876, 4151281, 4063042, 45769832, 4096042, 4221933, 4224879, 4096671, 4096670, 4224419, 4099652, 443733, 4096041,201826 |

**Supplementary table 2.** Baseline characteristics between high CV and low CV groups after the propensity score matching

| **Variables** | **Low CV**  **( n = 4,558 )** | **High CV**  **( n = 4,558 )** | ***p*** | **Absolute standardized difference** |
| --- | --- | --- | --- | --- |
| Age (years) | 62.8 ± 9.9 | 62.8 ± 9.9 | 1.00 | 0.00 |
| Male (n, %) | 2,253 (49.4) | 2,298 (50.4) | 0.35 | 0.02 |
| BMI (kg/m^2^) | 25.3 ± 7.8 | 25.1 ± 8.0 | 0.38 | 0.03 |
| Current smoker (n, %) | 338 (7.4) | 365 (8.0) | 0.29 | 0.02 |
| Alcohol drinking (n, %) | 386 (8.5) | 414 (9.1) | 0.30 | 0.02 |
| Systolic BP (mmHg) | 120.6 ± 10.0 | 121.0 ± 10.0 | 0.21 | 0.03 |
| Diastolic BP (mmHg) | 74.4 ± 5.5 | 74.6 ± 5.5 | 0.34 | 0.03 |
| Hypertension (n, %) | 3,043 (66.8) | 3,071 (67.4) | 0.53 | 0.01 |
| Anti-hypertensive drug (n, %) | 2,704 (59.3) | 2,658 (58.3) | 0.33 | 0.02 |
| Dyslipidemia (n, %) | 3,120 (68.5) | 3,229 (70.8) | 0.01 | 0.05 |
| Statin (n, %) | 2,055 (45.1) | 2,036 (44.7) | 0.69 | <.01 |
| TG-lowering drug (n, %) | 48 (1.1) | 66 (1.5) | 0.09 | 0.04 |
| Chronic kidney disease (n, %) | 943 (20.7) | 998 (21.9) | 0.16 | 0.03 |
| Insulin use (n, %) | 1,381 (30.3) | 1,418 (31.1) | 0.40 | 0.02 |
| Antiplatelet drug (n, %) | 1,851 (40.6) | 1,875 (41.1) | 0.61 | 0.01 |
| Anticoagulant (n, %) | 253 (5.6) | 316 (6.9) | <.01 | <.01 |
| Glucose (mg/dL) | 137.5 ± 33.8 | 140.2 ± 37.0 | <.01 | 0.08 |
| HbA1c (%) | 7.1 ± 1.2 | 7.1 ± 1.2 | 0.76 | <.01 |
| Creatinine (mg/dL) | 1.21 ± 1.24 | 1.28 ± 1.43 | 0.01 | 0.05 |
| hsCRP (mg/dL) | 3.2 ± 2.9 | 3.3 ± 2.9 | 0.09 | 0.04 |
| Total cholesterol (mg/dL) | 173.3 ± 28.2 | 173.8 ± 28.7 | 0.40 | 0.02 |
| LDL-cholesterol (mg/dL) | 104.9 ± 23.0 | 104.8 ± 23.8 | 0.90 | <.01 |
| HDL-cholesterol (mg/dL) | 48.0 ± 11.6 | 47.6 ± 11.2 | 0.19 | 0.03 |
| Triglyceride (mg/dL) | 144.5 ± 59.1 | 153.0 ± 60.7 | <.01 | 0.14 |
| TG-CV (%) | 21.2 ± 6.4 | 44.6 ± 13.5 | <.01 | 2.22 |
| TG-CEE (arbitrary unit) | 5,168.6 ± 1648.4 | 5,417.4 ± 1709.9 | <.01 | 0.15 |
| Number of TG measurement | 6.0 ± 3.9 | 6.1 ± 4.1 | 0.05 | 0.04 |
| Interval between TG measurement (day) | 231.4 ± 132.7 | 227.2 ± 131.6 | 0.14 | 0.03 |

Matching variables are age, sex, alcohol, smoking, hypertension, dyslipidemia, chronic kidney disease, creatinine, insulin use, TG-lowering drug, antiplatelet drug, anticoagulant, HbA1c, hsCRP, total cholesterol, LDL-cholesterol, HDL-cholesterol, triglyceride, number of TG measurement, interval between TG measurement. Categorical variables in n (%) and continuous variables in mean ± standard deviation. BMI, body mass index; BP, blood pressure; hsCRP, high-sensitivity C-reactive protein; LDL, low density lipoprotein; HDL, high density lipoprotein; TG-CV, coefficient of variation for serum TG level; TG-CEE, cumulative exposure estimates for serum TG level.

**Supplementary table 3.** Baseline characteristics between high CEE and low CEE groups after the propensity score matching

| **Variables** | **Low CEE**  **( n = 3,330 )** | **High CEE**  **( n = 3,330 )** | ***p*** | **Absolute standardized difference** |
| --- | --- | --- | --- | --- |
| Age (years) | 63.1 ± 9.8 | 63.1 ± 9.8 | 1.00 | 0.00 |
| Male (n, %) | 1,676 (50.3) | 1,681 (50.5) | 0.90 | <.01 |
| BMI (kg/m^2^) | 24.8 ± 7.4 | 25.7 ± 6.3 | <.01 | 0.14 |
| Current smoker (n, %) | 263 (7.9) | 279 (8.4) | 0.47 | 0.02 |
| Alcohol drinking (n, %) | 303 (9.1) | 317 (9.5) | 0.55 | 0.01 |
| Systolic BP (mmHg) | 120.8 ± 10.0 | 121.3 ± 10.0 | 0.13 | 0.05 |
| Diastolic BP (mmHg) | 74.4 ± 5.6 | 74.7 ± 5.4 | 0.03 | 0.07 |
| Hypertension (n, %) | 2,329 (69.9) | 2,322 (69.7) | 0.85 | <.01 |
| Anti-hypertensive drug (n, %) | 2,055 (61.7) | 2,066 (62.0) | 0.78 | <.01 |
| Dyslipidemia (n, %) | 2,431 (73.0) | 2,521 (75.7) | 0.01 | 0.06 |
| Statin (n, %) | 1,664 (50.0) | 1,588 (47.7) | 0.06 | 0.05 |
| TG-lowering drug (n, %) | 33 (1.0) | 51 (1.5) | 0.05 | 0.05 |
| Chronic kidney disease (n, %) | 704 (21.1) | 732 (22.0) | 0.40 | 0.02 |
| Insulin use (n, %) | 978 (29.4) | 995 (29.9) | 0.65 | 0.01 |
| Antiplatelet drug (n, %) | 1,428 (42.9) | 1,432 (43.0) | 0.92 | <.01 |
| Anticoagulant (n ,%) | 62 (1.9) | 73 (2.2) | 0.34 | 0.02 |
| Glucose (mg/dL) | 136.8 ± 34.3 | 139.6 ± 34.4 | <.01 | 0.08 |
| HbA1c (%) | 7.1 ± 1.2 | 7.1 ± 1.2 | 0.18 | 0.03 |
| Creatinine (mg/dL) | 1.25 ± 1.38 | 1.26 ± 1.37 | 0.77 | <.01 |
| hsCRP (mg/dL) | 3.2 ± 3.0 | 3.2 ± 2.8 | 0.71 | <.01 |
| Total cholesterol (mg/dL) | 171.1 ± 26.5 | 173.3 ± 24.8 | <.01 | 0.09 |
| LDL-cholesterol (mg/dL) | 104.2 ± 21.5 | 105.0 ± 23.4 | 0.17 | 0.03 |
| HDL-cholesterol (mg/dL) | 46.9 ± 10.6 | 46.3 ± 10.0 | 0.01 | 0.06 |
| Triglyceride (mg/dL) | 109.2 ± 21.1 | 193.8 ± 58.0 | <.01 | 1.94 |
| TG-CV (%) | 29.9 ± 14.0 | 36.0 ± 18.3 | <.01 | 0.37 |
| TG-CEE (arbitrary unit) | 4,181.1 ± 593.6 | 6,551.3 ± 1585.6 | <.01 | 1.94 |
| Number of TG measurement | 6.2 ± 4.4 | 6.2 ± 4.2 | 0.97 | <.01 |
| Interval between TG measurement (day) | 229.2 ± 131.7 | 230.6 ± 133.5 | 0.67 | 0.01 |

Matching variables are age, sex, alcohol, smoke, hypertension, dyslipidemia, chronic kidney disease, creatinine, insulin use, TG-lowering drug, antiplatelet drug, anticoagulant, HbA1c hsCRP, total cholesterol, LDL-cholesterol, HDL-cholesterol, number of TG measurement, interval between TG measurement, TG-CV. Categorical variables in n (%) and continuous variables in mean ± standard deviation. BMI, body mass index; BP, blood pressure; hsCRP, high-sensitivity C-reactive protein; LDL, low density lipoprotein; HDL, high density lipoprotein; TG-CV, coefficient of variation for serum TG level; TG-CEE, cumulative exposure estimates for serum TG level.

**Supplementary table 4.** Incidence of endpoint in the full population before propensity score matching

|  | **Low CV (n = 12,967)** | **High CV (n=12,966)** | **p-value** | **Low CEE (n= 12,967)** | **High CEE (n= 12,966)** | **p-value** |
| --- | --- | --- | --- | --- | --- | --- |
| MAE | **900 (6.9)** | **1022 (7.9)** | **<0.01** | **1004 (7.7)** | **918 (7.1)** | **0.04** |
| New-onset MI | **196 (1.5)** | **272 (2.1)** | **<.01** | 240 (1.9) | 228 (1.7) | 0.58 |
| New-onset stroke | **203 (1.6)** | **254 (2.0)** | **0.02** | 569 (4.4) | 556 (4.3) | 0.69 |
| All-cause death | **203 (1.57)** | **254 (2.0)** | **0.02** | **264 (2.0)** | **193 (1.5)** | **<.01** |

Values are presented as number of incidence (%). MAE, major adverse event; MI, myocardial infarction. p-value for the log-rank test.

**Supplementary table 5.** Cox regression analysis for TG CV in the full population before propensity score matching

|  | **Crude model** | **Model 1** | **Model 2** | **Model 3** | **Model 4** |
| --- | --- | --- | --- | --- | --- |
| MAE | **1.14**  **(1.04-1.25)** | **1.22**  **(1.12-1.34)** | **1.21**  **(1.10-1.32)** | **1.17**  **(1.07-1.28)** | **1.18**  **(1.07-1.31)** |
| New-onset MI | **1.39**  **(1.16-1.67)** | **1.44**  **(1.20-1.73)** | **1.42**  **(1.18-1.71)** | **1.30**  **(1.11-1.60)** | **1.29**  **(1.06-1.58)** |
| New-onset stroke | 1.00  (0.89-1.12) | 1.07  (0.95-1.20) | 1.06  (0.94-1.19) | 1.04  (0.92-1.17) | 1.03  (0.91-1.18) |
| All-cause death | **1.25**  **(1.04-1.51)** | **1.39**  **(1.15-1.67)** | **1.36**  **(1.13-1.63)** | **1.33**  **(1.11-1.61)** | **1.38**  **(1.11-1.72)** |

Values are presented as hazard ratio (95% confidence interval). Model 1 adjusted for age and sex. Model 2 adjusted for Model 1 plus alcohol and smoking. Model 3 adjusted for Model 2 plus hypertension, dyslipidemia, and chronic kidney disease. Model 4 adjusted for Model 3 plus total cholesterol, LDL-C, HDL-C, number of TG measurement, and interval between TG measurements.

**Supplementary table 6.** Cox regression analysis for TG CEE in the full population before propensity score matching

|  | **Crude model** | **Model 1** | **Model 2** | **Model 3** | **Model 4** |
| --- | --- | --- | --- | --- | --- |
| MAE | 0.91  (0.83-1.00) | 0.99  (0.90-1.08) | 0.99  (0.90-1.08) | **0.89**  **(0.81-0.97)** | 0.93  (0.83-1.04) |
| New-onset MI | 0.95  (0.79-1.14) | 1.00  (0.83-1.20) | 1.00  (0.83-1.20) | **0.79**  **(0.66-0.95)** | 0.86  (0.69-1.07) |
| New-onset stroke | 0.98  (0.87-1.10) | 1.06  (0.94-1.19) | 1.05  (0.94-1.19) | 0.98  (0.87-1.10) | 0.96  (0.83-1.12) |
| All-cause death | 0.73  (0.61-0.88) | 0.83  (0.69-1.01) | 0.83  (0.68-1.00) | **0.79**  **(0.65-0.95)** | 0.91  (0.72-1.15) |

Values are presented as hazard ratio (95% confidence interval). Model 1 adjusted for age and sex. Model 2 adjusted for Model 1 plus alcohol and smoking. Model 3 adjusted for Model 2 plus hypertension, dyslipidemia, and chronic kidney disease. Model 4 adjusted for Model 3 plus total cholesterol, LDL-C, HDL-C, number of TG measurement, and interval between TG measurements.
